# Hungarian Adaptation and Validation of the Short Food Literacy Questionnaire (SFLQ-HU)

**DOI:** 10.3390/nu17111796

**Published:** 2025-05-26

**Authors:** Viola Keczeli, Andrea Gubicskóné Kisbenedek, Alexandra Makai, Zsófia Verzár

**Affiliations:** 1 Doctoral School of Health Sciences, Faculty of Health Sciences, University of Pécs, 7621 Pécs, Hungary; andrea.kisbenedek@etk.pte.hu (A.G.K.); verzar.zsofia@pte.hu (Z.V.); 2 Institute of Nutritional Sciences and Dietetics, Faculty of Health Sciences, University of Pécs, 7621 Pécs, Hungary; 3 Institute of Physiotherapy and Sport Sciences, Faculty of Health Sciences, University of Pécs, 7621 Pécs, Hungary; alexandra.makai@etk.pte.hu

**Keywords:** food literacy, health literacy, questionnaire validation, young adults, Short Food Literacy Questionnaire

## Abstract

Background: Food literacy (FL) plays a crucial role in promoting healthy eating and sustainable food choices. The Short Food Literacy Questionnaire (SFLQ) has been widely used to assess FL in different populations. Objective: This study aimed to adapt and validate the SFLQ in Hungarian (SFLQ-HU) and evaluate its psychometric properties among university students. Methods: The adaptation followed international guidelines, including forward- and back-translation, pre-testing, and confirmatory factor analysis (CFA). Reliability was assessed using Cronbach’s alpha, while criterion validity was tested through correlations with other health-related scales. For statistical analysis, IBM AMOS 26.0 and SPSS 28.0 software were used. Results: The survey involved 1325 (*n* = 1325) young adults in 2024. Overall, 76% of the sample were women (*n* = 1007), and 77.43% of the sample were under 25 years old (*n* = 1025). The FL level scored 36.47 points (SD: 7.53), with the highest score being 52 and the lowest score being 14.8. The SFLQ-HU demonstrated excellent internal consistency (Cronbach’s α = 0.850) and a good fit for the one-factor structure in CFA. Test–retest reliability confirmed the stability of the instrument. Significant correlations were found between the SFLQ-HU and measures of health literacy (r = 0.197; *p* < 0.001), food choice motivations (r = 0.394; *p* < 0.001), and barriers to health behaviors (r = 0.353; *p* < 0.001). Conclusions: The SFLQ-HU is a reliable and valid tool for assessing food literacy of young Hungarian-speaking adults. It can facilitate future research and public health initiatives aimed at improving FL and promoting healthy dietary habits.

## 1. Introduction

Food literacy (FL) is a complex and multifaceted concept encompassing knowledge, skills, and behaviors related to food and nutrition [1]. It refers to an individual’s ability to acquire, interpret, and apply food and nutrition information to promote health [2]. According to Benn, FL also includes the capacity to critically evaluate messages about food, make informed food choices, and understand the cultural, social, and personal factors that influence dietary habits [3]. FL extends beyond individual health behaviors; it covers the entire food production chain, including where and how food is produced, who benefits or loses from food purchases, who has access to food, and how food waste is managed. This broader perspective integrates cultural, environmental, social, and economic dimensions, aligning with national policies aimed at sustainable food systems [4].

Assessing FL in diverse populations has gained increasing recognition as a crucial factor in public health research. Research has demonstrated its potential role in addressing complex health challenges, ranging from obesity to environmental sustainability [5]. Several studies highlight that non-communicable diseases, such as obesity and cardiovascular conditions, are strongly associated with dietary habits [6,7]. In Hungary, obesity is an escalating public health concern. The 2019 National Nutrition and Diet Status Survey reported that 34% of Hungarian adults are overweight, and another 34% are classified as obese. Additionally, the 2021 Organisation for Economic Co-operation and Development (OECD) report ranked Hungary as the second most obese country in the European Union, with 35.4% of adults being overweight and 24.5% obese [8,9]. According to Eurostat, 53% (17% obese) of the adult population in the European Union was overweight in 2019 [10]. In their 2023 study, Meng et al. pointed out that the prevalence of obesity in Germany doubled between 1987 and 2016, accounting for more than a quarter of German adults, with both men and women having an average body mass index (BMI) of over 26 kg/m^2^ [11]. Research on FL is still relatively new, originating in the early 2000s in the United States before expanding globally.

In Hungary, no validated FL scale or extensive study on the topic exists, and the concept has not yet been formally adapted into Hungarian. To measure FL, the Short Food Literacy Questionnaire (SFLQ) was selected, a 12-item instrument recognized as one of the most widely used tools, as indicated in a 2018 review [12]. The Polish adaptation of the SFLQ references a study by Yuen et al., emphasizing the questionnaire’s ability to measure functional, interactive, and critical elements of FL. The original SFLQ was developed by Krause et al. in Switzerland in 2016, primarily as part of a larger initiative to reduce salt intake in the Swiss adult population. The authors confirmed that the instrument had a one-factor latent structure and demonstrated adequate construct validity [12,13]. The SFLQ is a concise, practical tool designed for public health applications. Although originally developed for Switzerland, it has been successfully adapted for other populations. While FL consists of multiple components, this questionnaire primarily focuses on skills and abilities for making healthy food choices. Since its initial development, the SFLQ has been validated in several languages and cultural contexts.

In 2019, Durmuş et al. validated the SFLQ in Turkish, emphasizing the public health significance of assessing FL levels among adults. Durmuş et al. reported a moderate positive correlation between FL scores from the SFLQ and health literacy scores derived from the Turkey Health Literacy Scale-32 (TSOY-32) [14]. Additionally, a study among Turkish university students revealed that higher SFLQ scores were significantly associated with better general health perceptions and a habit of reading food labels [14]. The role of the questionnaire validated in Portuguese in 2022 was the same as that of the questionnaire adapted to Hungarian, allowing Brazilian researchers to compare their results using an internationally validated questionnaire [15]. In Italy, Sará and Trieste validated the SFLQ on an adult sample, finding significant correlations between FL and shopping behavior, gender, and place of residence [16]. Similarly, Itzkovitz et al. (2022) found a strong association between higher SFLQ scores and improved cooking skills among young adults with type 1 diabetes in Canada [17]. In 2022, Zwierczyk et al. culturally adapted and validated the Polish version of the SFLQ, confirming its good internal consistency. Unlike previous studies, they identified a three-factor latent structure, distinguishing between “access to information”, “knowledge”, and “information evaluation”. Their confirmatory factor analysis (CFA) indicated a strong fit for this model, making the Polish version a reliable tool for assessing educational interventions and determinants of FL [18].

The aim of this study was to adapt and validate the SFLQ in Hungarian, assessing its reliability, construct validity, and applicability among university students in Hungary. Validating the Hungarian version of the SFLQ will facilitate further research on FL levels in Hungarian-speaking populations, fostering cross-cultural comparisons and supporting targeted public health interventions.

## 2. Materials and Methods

### 2.1. The Short Food Literacy Questionnaire Features (SFLQ)

The Short Food Literacy Questionnaire (SFLQ) was developed to assess key competencies related to food literacy, including functional, interactive, and critical dimensions. Krause et al. designed the SFLQ as part of a broader initiative to reduce salt consumption in the Swiss population [13,19,20]. The measurement properties of the questionnaire were evaluated using exploratory factor analysis, which confirmed its unidimensional structure. The final version of the SFLQ consists of 12 items, rated on a four- or five-point Likert scale, covering essential components of nutrition literacy and food literacy. The total score ranges from 7 to 52, with higher scores indicating greater food literacy. The internal consistency of the questionnaire, measured by Cronbach’s alpha, was reported as 0.82, demonstrating good reliability.

### 2.2. The Brief Health Literacy Screening Tool (BRIEF)

To evaluate external convergent validity, health literacy levels were assessed alongside sociodemographic characteristics, dietary habits, and food choices. Health literacy was measured using the Brief Health Literacy Screening Tool (BRIEF), which was validated in Hungarian by Mátyás et al. in 2021 [21]. The BRIEF questionnaire consists of four self-administered questions and serves as a practical tool for subjectively assessing health literacy. Responses are rated on a five-point Likert scale (1–5), and the total score ranges from 4 to 20. Based on this score, health literacy is categorized as inadequate (4–12), marginal (13–16), or adequate (17–20).

### 2.3. The Food Choice Questionnaire (FCQ)

To assess dietary habits, self-constructed questions were used, while factors influencing food choices were evaluated using the Food Choice Questionnaire (FCQ), which was validated in Hungarian by Szakály et al. in 2018 [22]. The 36-item questionnaire, originally developed by Steptoe et al., categorizes food choice motivations into seven main factors, which, according to the authors, play a crucial role in determining food selection:

1. Health and ingredients—The health effects of foods and factors related to ingredients.
2. Mood—The impact of food consumption on mood and emotional state.
3. Convenience—Ease and speed of food preparation and related preferences.
4. Price and availability—The price of food and its ease of availability.
5. Sensory impressions—The appearance, smell, taste, texture, and other sensory characteristics of food.
6. Familiarity and tradition. Foods that are already familiar and their personal and cultural significance (e.g., childhood memories).
7. Ethical and sustainability considerations—Consideration of the environmental, ethical, and sustainability factors of food.

The participants rated the importance of these factors on a 0-to-5 Likert scale, reflecting the extent to which each aspect influences their food purchasing behavior. For the analysis, the mean scores for each motivational factor were examined and compared.

### 2.4. The Motivators and Barriers of Health Behaviors Model (MBHB Model)

The MBHB model (Motivators and Barriers of Health Behaviors) is a theoretical framework designed to examine the motivations and barriers influencing health behaviors, making it particularly relevant for university students. Originally developed by Rosenstock in 1974 and later refined by Downes in 2008, the model has been applied in various health-related contexts [23,24]. In 2020, Papp-Bata utilized the MBHB model to analyze consumption habits and food preferences, emphasizing its key principle: “The MBHB model assumes that individuals for whom motivations are more important than barriers are more likely to engage in positive health behaviours to achieve desired health outcomes than those who have insurmountable barriers and are more likely to be constrained in engaging in positive health behaviours [25,26].” The MBHB model measures barriers and motivators to health behavior on a scale of seven statements (1 = strongly disagree, 4 = strongly agree, 0 = do not know/no answer). The MBHB model is assessed by comparing the average score of barriers and motivators.

The overall MBHB score is determined by comparing the average scores of motivators and barriers:

- A higher motivator score suggests that the individual perceives more supportive factors for maintaining a healthy lifestyle.
- A higher barrier score indicates a greater likelihood of adopting unhealthy lifestyle and dietary habits due to perceived obstacles.

This model provides valuable insights into the psychosocial determinants of health behaviors, particularly in the context of dietary habits, making it an essential component of the present study.

### 2.5. The Process of Validation

The validation of the Hungarian version of the Short Food Literacy Questionnaire (SFLQ) followed the six-step guidelines outlined by Beaton et al. and the COSMIN guideline [27,28]. The process included translation, synthesis, back-translation, pre-testing, internal consistency assessment, and external convergent validity testing. As a first step, the research team sought written permission and support from the authors of the original English-language questionnaire for the questionnaire they wanted to validate. Subsequently, the 12-question questionnaire was translated into Hungarian. The translation was carried out by two independent persons, one of whom was a qualified medical translator and one of whom was a qualified medical translator with a medical degree. From the two Hungarian translations, a synthesis was created by the research committee, which included the form considered most applicable to the given question/statement (members: medical doctor, dietician, epidemiologist, sociologist, and language teacher). Next, the synthesis was reverse-engineered by two independent translators, one a professional translator and the other a health professional with a higher-level degree in languages. The synthesis was considered to be final when the reverse translators certified, in writing, that the synthesis, when translated back into English, did not differ in content, form, and quality from the original English questionnaire. In consultation with the original authors, changes to the questionnaire were necessary to adapt it to the Hungarian population. Such a change was made in the case of question 3: *How familiar are you with the Swiss Food Pyramid?* Instead, question 3 became *How familiar are you with the OKOSTÁNYÉR^®^ recommendation*? (OKOSTÁNYÉR^®^ is the latest Hungarian nutritional recommendation from the National Association of Hungarian Dietitians.) [29]. A similar adaptation was made for the Hungarian population for questions 4 and 5. Question 4, i.e., *I know the official Swiss recommendations about fruit and vegetable consumption*, was adapted as follows: *I know the official Hungarian recommendations on fruit and vegetable consumption.* Question 5, i.e., *I know the official Swiss recommendations about salt intake*, was adapted as follows: *I know the official Hungarian recommendations about salt intake.*

Subsequently, a pre-testing of the food literacy questionnaire was conducted with 50 participants in order to test the applicability of the questionnaire on a native Hungarian-speaking sample of appropriate age, i.e., university students. The problematic/interpretation-confusing terms indicated during the pre-testing were corrected after the pre-testing (e.g., use of the Hungarian recommendation according to Hungarian standards). The resulting final format of the food literacy questionnaire was proposed for the next level of validation, which is the internal consistency test. The participants were asked to complete the questionnaire and then asked to complete it again after 3 weeks. A difference-in-difference test was conducted between the two surveys to show that there was no significant difference between the first and second questionnaire responses. Subsequently, data collection was carried out with the developed Hungarian adaptation.

### 2.6. Statistical Analysis

After the data collection, statistical analyses were conducted to assess the reliability, validity, and structural properties of the Hungarian version of the SFLQ.

#### 2.6.1. Reliability Analysis

To assess the reliability of the Hungarian version of the Short Food Literacy Questionnaire (SFLQ-HU), internal consistency and test–retest reliability analyses were conducted.

#### 2.6.2. Internal Consistency

Internal consistency was evaluated using Cronbach’s alpha (α), which measures the degree of interrelatedness among the questionnaire items. A Cronbach’s alpha value between 0.70 and 0.95 was considered adequate, indicating acceptable homogeneity within the scale [30].

#### 2.6.3. Test–Retest Reliability

The stability of the SFLQ over time was assessed using the Intraclass Correlation Coefficient (ICC). The questionnaire was administered to a subset of participants (*n* = 35) twice, with a three-week interval between completions. ICC values were interpreted as follows:

- ≥0.75–1.00: excellent reliability;
- 0.60–0.74: good reliability;
- 0.40–0.59: moderate reliability;
- ≤0.39: poor reliability.

An ICC value of ≥0.75 was considered an indication of excellent test–retest reliability [31].

#### 2.6.4. Validity

##### Structural Validity

Structural validity was explored by score distributions and floor and ceiling effects; the latter was considered present if more than 15% of the sample achieved the lowest (floor) or highest (ceiling) possible score. To examine the factor structure of the SFLQ, confirmatory factor analysis (CFA) was conducted using maximum likelihood estimation [32]. Model fit was evaluated based on standard goodness-of-fit indices, including the following:

- Chi-square test (χ^2^/*df*): Values < 3 were considered an acceptable model fit.
- Comparative Fit Index (CFI) and Tucker–Lewis Index (TLI): Values ≥ 0.90 indicated an acceptable fit, while values ≥ 0.95 indicated a good fit.
- Root Mean Square Error of Approximation (RMSEA): Values ≤ 0.08 indicated an acceptable fit, while values ≤ 0.06 indicated a good fit.

##### Convergent Validity

To determine whether the Hungarian SFLQ measures food literacy in a meaningful way, Pearson’s correlation coefficients were calculated to examine its relationship with the Brief Health Literacy Screening Tool (BRIEF), the MBHB Model, and the Food Choice Questionnaire (FCQ). A significant positive correlation between SFLQ and BRIEF scores was expected, indicating a strong relationship between food literacy and health literacy. Similarly, a positive correlation with FCQ motivational factors (e.g., health, sustainability, price) was hypothesized [33].

##### Discriminant Validity

For the examination of discriminant validity, based on the students’ science field, two groups of students were created (students studying the medical and health science field vs. students studying the non-medical and health science field). The Mann–Whitney U-test was used to test for discriminant validity to examine differences in SFLQ—HU scores between the students in the medical and health science field and the non-medical and health science field.

Descriptive statistics were applied to establish sample characteristics, central tendency estimates, and variability in the dataset. These data are presented as means, standard deviations (SDs), or percentages (%). All statistical analyses were performed using IBM SPSS Statistics 28.0 and IBM Amos 26.0. A *p*-value of <0.05 was considered statistically significant.

### 2.7. Selection of Participants and Sample Characteristics

**Figure 1:**
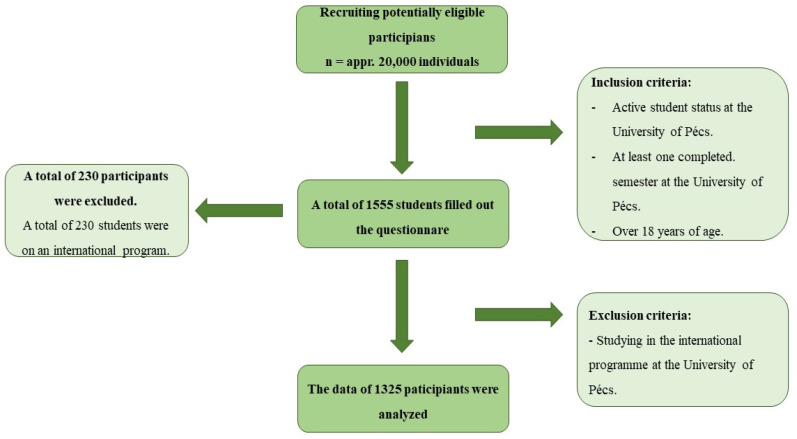
Flowchart illustrating the recruitment and data collection process for the Hungarian validation of the SFLQ-HU.

The participants for our cross-sectional study were recruited non-randomly among the students of the University of Pécs in the 2023/2024 academic year by convenience sampling (Figure 1).

The inclusion criteria included being at least 18 years of age, having completed at least one semester at the university, and being an active student in a bachelor’s or master’s degree program in a departmental program, higher education vocational training, or further education.

The exclusion criteria included participation in an international program, i.e., a legal status in a non-Hungarian language course, and lack of motivation and consent. The sample thus consisted of 1325 students. The number of items was determined considering the rule of at least 10 participants per item (question) [34].

### 2.8. Ethical Compliance

Participation in this research was voluntary for the students, and anonymity was assured. Ethical guidelines were followed in all segments of this research. The prepared database was treated confidentially and was used only for the preparation of this research. The European Union GDPR regulations were adhered to throughout this research. This research complies with the principles of the Helsinki Declaration. It is not possible to identify the students based on the questions. Permission to conduct this research among the students of the University of Pécs was granted by the Regional Research Ethics Committee of the Clinical Centre of the University of Pécs. According to the decision of the Committee’s meeting of 15 December 2023, the ethical approval number 9760-PTE2023 was issued.

**Figure 2:**
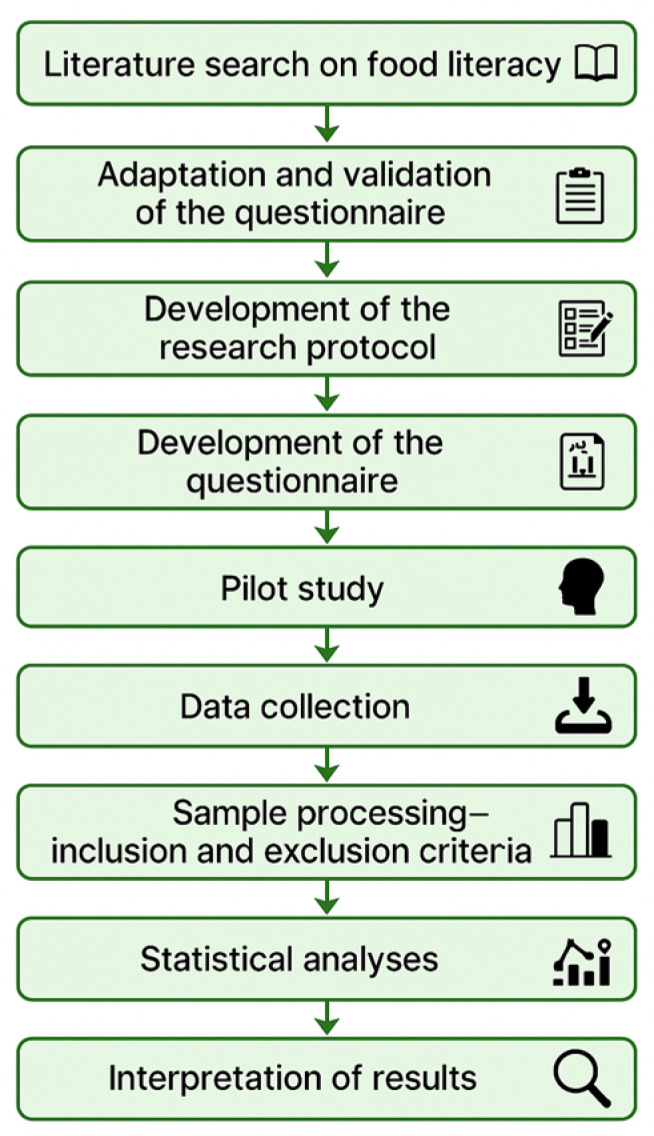
Overview of the research process (This figure was created using ChatGPT version 4o).

Figure 2 presents a visual summary of the complete research process carried out in the current study. The diagram outlines each key phase of the methodology, from the initial literature review and questionnaire adaptation to data collection and statistical analysis. This step-by-step representation aims to enhance the reproducibility and transparency of this study and aligns with the reviewer’s request for a comprehensive overview.

## 3. Results

The following section presents the key findings from the statistical analyses, providing evidence for the reliability and validity of the SFLQ-HU. Considering the inclusion and exclusion criteria, our sample eventually consisted of 1325 students in total. In terms of the sex ratio, our sample consisted of 318 male students (*n* = 318; 24%), representing less than a quarter of the sample, and 1007 female (*n* = 1007; 76%) students. The mean age of the undergraduate students was 24.71 years (SD: 8.51). In terms of place of residence, the majority lived in a larger municipality, 119 students (8.98%) lived in the capital, and 439 students (33.13%) lived in a city in a county seat. Students from all 10 faculties of the University of Pécs participated in the survey, with the highest proportion of students from the Faculty of Health Sciences, who represented almost 40% of our sample (*n* = 510; 38.49%). The whole sample’s profile and sociodemographic characteristics are presented in Table 1.

The internal consistency of the SFLQ—HU was excellent with a Cronbach’s α of 0.85 (Table 2). The test–retest reliability of the SFLQ—HU in a sample of 50 participants yielded excellent results (ICC: 0.921; 95% CI: 0.799–0.969).

**Figure 3:**
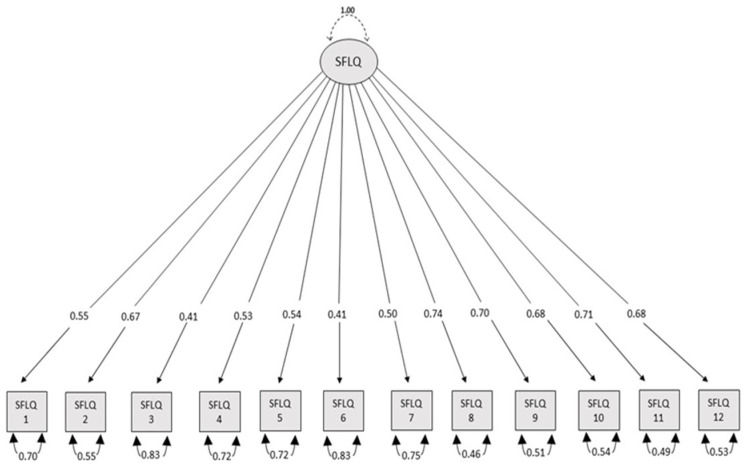
Confirmatory factor analysis (CFA) results for the Hungarian version of the Short Food Literacy Questionnaire (SFLQ-HU).

The confirmatory factor analysis (CFA) supported the one-factor structure of the Hungarian version of the Short Food Literacy Questionnaire (SFLQ-HU), demonstrating good model fit (RMSEA = 0.043; TLI = 0.973; CFI = 0.980; χ^2^ = 170.095, *df* = 50, *p* < 0.001; χ^2^/*df* = 3.402). All factor loadings were significant (*p* < 0.01), ranging from 0.41 to 0.74, supporting the construct validity of the scale. The applied modification indices between the different items are presented in Figure 3.

Table 3 shows the correlation between the SFLQ—HU and the other measures. Spearman’s rank correlation analysis revealed significant associations between the SFLQ-HU total score and various constructs related to health behavior, food choice motivations, and health literacy. The strongest correlation was observed with the FCQ first factor (r = 0.394, *p* < 0.01), indicating a notable link between food literacy and health-related food choices. Additionally, significant correlations were found with the MBHB model motivators (r = 0.314, *p* < 0.01) and barriers (r = −0.353, *p* < 0.01), further supporting the construct validity of the SFLQ-HU. Weak to moderate correlations were identified with other FCQ factors and the BRIEF questionnaire.

To examine discriminant validity, we applied the Mann–Whitney U test. The results indicated a significant difference in the mean scores of the SFLQ-HU between the students from different scientific fields. The students’ average score for food literacy was 36.47 (SD: 7.53), with the highest being 52 and the lowest 14.8. The students in the medical and health sciences group (38.18 ± 7.28) had significantly higher SFLQ-HU scores than the students in non-medical and health sciences fields (33.53 ± 7.04) (*p* < 0.001). The relationship between their mothers’ educational attainment and the overall SFLQ-HU score did not reach statistical significance (*p* = 0.229). However, when the mean score for question 2 was examined, which assesses participants’ ability to evaluate nutrition-related information, a significant association was observed (*p* = 0.044). This result suggests that the students whose mothers had higher educational attainment were better at evaluating and interpreting nutrition information. The difference in the SFLQ-HU total scores between the male and female students was examined using the Mann–Whitney U test. The results revealed a significant difference between the two groups (U = 178,976.500, *p* = 0.002). The female students had higher SFLQ-HU scores (Mean Rank = 681.73) compared to the male students (Mean Rank = 603.68), indicating better food literacy levels among the females.

## 4. Discussion

The validation process and subsequent analyses revealed important insights into the psychometric properties of the SFLQ-HU, with several findings aligning with international studies. The aim of this study was to adapt and validate the Short Food Literacy Questionnaire (SFLQ) in Hungarian, assessing its reliability and validity among university students.

The SFLQ has been widely used in previous studies to measure the food literacy of the adult population and has been validated from the original German version, which was later published in English by the original authors, into a number of languages, including Polish, Italian, Portuguese, and Turkish [14,15,16,18,19]. We examined the psychometric properties of the Hungarian version (SFLQ-HU), following a cross-cultural adaptation and validation process consistent with the methodologies described in the relevant literature. The reliability and validity of the SFLQ-HU aligned with the findings of similar validation studies. Consistent with previous research, our confirmatory factor analysis (CFA) supported the one-factor structure of the SFLQ-HU. Furthermore, the deletion of individual items did not significantly affect Cronbach’s alpha, indicating the robustness of the scale. Similar to the Turkish validation study, no significant floor or ceiling effects were observed [14]. The SFLQ-HU demonstrated strong psychometric properties, exhibiting good reliability and validity. The internal consistency was excellent (Cronbach’s α = 0.850), closely matching the values reported in other validations, including the Turkish (Cronbach’s α = 0.803), Polish (Cronbach’s α = 0.841), Italian (Cronbach’s α = 0.80), Brazilian (McDonald’s ω = 0.874), and original German (Cronbach’s α = 0.820) versions [13,14,15,16,18]. Additionally, the SFLQ-HU showed excellent test–retest reliability, further confirming its stability over time. A 3-week interval was used between the initial test and the retest, as suggested by the relevant literature [35,36]. Criterion validity was confirmed in this study. The SFLQ-HU was significantly and negatively associated with measures of MBHB model barriers and the third factor of FCQ. The SFLQ—HU was significantly and positively associated with measures of the MBHB model motivators, the total score of BRIEF, and the first, sixth, and eighth factors of FCQ. The correlation between the SFLQ-HU and the total score of BRIEF (r = 0.197) was in line with the results from the Turkish validation study (r = 0.513), while the Polish study also highlighted a positive relationship without specifying a correlation coefficient.

In this research, we also found that higher levels of food literacy are associated with higher levels of health literacy, consistent with the results of other cross-cultural adaptations and validations [14,15]. Our findings regarding the relationship between the mothers’ educational attainment and food literacy were partially consistent with previous studies. Although the overall SFLQ-HU score did not show a significant association with the mothers’ level of education (*p* = 0.229), a deeper analysis revealed a significant relationship for question 2, which specifically addresses the ability to evaluate nutrition-related information (*p* = 0.044). This indicates that students whose mothers had higher educational qualifications were more confident and capable of evaluating and interpreting nutrition information. This result aligns with research that emphasizes the role of the family environment and parental education in developing critical health-related skills. Studies in Turkey and Italy have similarly pointed out that higher parental education is associated with greater health awareness and improved food literacy among university students [14,15,16]. Durmuş et al. found a moderate correlation between health literacy and food literacy in their Turkish university sample, with participants from more educated families showing a better understanding of complex food labels and health-related information. Similarly, in Sará and Trieste’s Italian validation, gender and socioeconomic background were significant predictors of shopping behavior and food literacy.

The cross-cultural comparison highlights both similarities and differences in the SFLQ adaptations. While the Turkish and Italian studies confirmed the questionnaire’s one-factor structure, the Polish validation identified a three-factor structure, distinguishing between “access to information”, “knowledge”, and “information evaluation”. This discrepancy may reflect cultural differences in how respondents process and evaluate food-related information. In Hungary, our findings aligned more closely with the Turkish and Italian results, supporting a single underlying construct of food literacy. Furthermore, the Italian study identified significant correlations between food literacy scores and variables such as gender and place of residence, with urban students generally scoring higher than their rural counterparts. This study identified significant gender differences in food literacy levels, with female young adults scoring higher on the SFLQ-HU compared to their male peers. This finding is consistent with previous research, including the Turkish validation study, which also reported higher food literacy scores among female participants [14]. The gender gap in food literacy has been attributed to several factors, including differences in health awareness, information-seeking behavior, and nutritional knowledge. Research suggests that women are generally more involved in food-related activities, such as meal preparation and grocery shopping, which may contribute to their higher food literacy levels. In contrast, male students may have less exposure to practical food-related skills and less interest in nutrition information, potentially explaining their lower scores. This finding highlights the importance of developing gender-sensitive educational interventions to improve food literacy among all students. These findings underscore the importance of considering cultural and contextual factors in food literacy research. While the overall SFLQ-HU score did not correlate significantly with the mothers’ educational level, specific components of food literacy were indeed influenced. This suggests that targeted educational interventions focusing on critical thinking and information evaluation skills may be especially beneficial for students from less privileged backgrounds. As highlighted in previous studies, improving these skills can help bridge the gap in health and food literacy, ultimately leading to healthier lifestyle choices. The strengths of this study include its large number of elements. The limitations of this study include the fact that it was conducted on university students, i.e., young adults, and is therefore not representative of the Hungarian population.

## 5. Conclusions

This study aimed to provide a validated tool for measuring food literacy in Hungarian-speaking populations, contributing to the growing body of research in this field. The Hungarian adaptation and validation of the Short Food Literacy Questionnaire (SFLQ-HU) demonstrated strong psychometric properties with excellent reliability and validity. The confirmatory factor analysis (CFA) supported the one-factor structure of the questionnaire, consistent with previous validations in other languages. The internal consistency was high, and the test–retest reliability confirmed the stability of the questionnaire over time.

The findings revealed significant associations between food literacy levels and sociodemographic factors, such as gender and field of study. Female students and those in medical and health sciences achieved higher scores in food literacy compared to their counterparts. Interestingly, while the total SFLQ-HU score was not significantly associated with the mothers’ educational attainment, question-specific analysis showed that students with mothers holding higher educational qualifications were more confident in evaluating nutrition-related information.

These results suggest that the SFLQ-HU is a reliable and valid tool for assessing food literacy in Hungarian-speaking populations. The SFLQ-HU makes Hungarian and international food literacy data comparable, thus allowing for a better interpretation of the results and the development of common improvement strategies.

## Figures and Tables

**Table 1:** Sample characteristics (*N* = 1325).

Variable	Absolute Frequency *n* = 1325	Relative Frequency %
**Gender**		
Male	318	24.00%
Female	1007	76.00%
**Age Group**		
25 years old or younger	1026	77.43%
Over 25 years old	299	22.57%
**Type of residence**		
Village	353	26.64%
Town (not county seat)	414	31.25%
County seat	439	33.13%
Capital city	119	8.98%
**Household type**		
Living with parents	484	36.53%
Living alone in a rented apartment	76	5.74%
Living alone in own property	62	4.68%
Living in a rented apartment with roommate	192	14.49%
Living in a dormitory	203	15.32%
Living with partner/spouse	306	23.09%
Other	2	0.15%
**Faculty at PTE**		
Faculty of Law and Political Sciences	48	3.62%
Faculty of General Medicine	303	22.87%
Faculty of Humanities and Social Sciences	145	10.94%
Faculty of Health Sciences	510	38.49%
Faculty of Pharmacy	27	2.04%
Faculty of Business and Economics	100	7.55%
Faculty of Cultural Sciences, Education and Regional Development	44	3.32%
Faculty of Engineering and Information Technology	63	4.75%
Faculty of Arts	24	1.81%
Faculty of Science	61	4.6%
**Year of study**		
First	659	49.74%
Second	288	21.74%
Third	195	14.72%
Fourth	119	8.98%
Fifth	28	2.11%
Sixth	36	2.72%
**Mode of study**		
Full-time	1091	82.34%
Part-time	234	17.66%
**Education level**		
Bachelor (BSc)	752	56.75%
Undivided program	363	27.40%
Master (MSc)	94	7.09%
Doctoral (PhD)	62	4.68%
Postgraduate specialization	33	2.49%
Higher vocational training	21	1.58%
**Mother’s highest educational level**		
Primary school	59	4.45%
Secondary education	648	48.91%
College/university	618	46.64%

**Table 2:** Internal consistency of the SFLQ—HU (*n* = 1325).

Item	Mean	SD	Corrected Item-Total Correlation	Cronbach’s α When Deleted
SFLQ—HU Item 1	3.25	0.67	0.476	0.843
SFLQ—HU Item 2	4.12	0.64	0.608	0.838
SFLQ—HU Item 3	3.11	1.66	0.442	0.858
SFLQ—HU Item 4	2.58	1.14	0.593	0.833
SFLQ—HU Item 5	2.52	1.16	0.583	0.834
SFLQ—HU Item 6	3.03	0.93	0.363	0.849
SFLQ—HU Item 7	3.36	1.03	0.447	0.844
SFLQ—HU Item 8	3.86	0.90	0.645	0.831
SFLQ—HU Item 9	2.46	0.98	0.609	0.833
SFLQ—HU Item 10	2.41	0.97	0.574	0.835
SFLQ—HU Item 11	2.92	0.83	0.605	0.835
SFLQ—HU Item 12	2.79	0.89	0.599	0.834
SFLQ—HU Cronbach’s α	**0.850**			

**Table 3:** Convergent validity of the SFLQ-HU and the other measurement tools (*N* = 1325).

Questionnaire	Spearman’s Rank Correlation Coefficients with the Total Score of the SFLQ-HU (r. *p*) (*n* = 1325)
MBHB model motivators	0.314 **
MBHB model barriers	−0.353 **
BRIEF total score	0.197 **
FCQ first factor—Health and ingredients	0.394 **
FCQ second factor—Mood	0.064 *
FCQ third factor—Convenience	−0.097 **
FCQ fourth factor—Price and availability	−0.073 ***
FCQ fifth factor—Sensory impressions	0.117 **
FCQ sixth factor—Familiarity and tradition	−0.052
FCQ seventh factor—Ethical and sustainability considerations	0.206 **
Level of significance. * *p* = 0.031; ** *p* < 0.001; *** p = 0.012	

## Data Availability

The original contributions presented in this study are included in the article. Further inquiries can be directed to the corresponding author.

## Abbreviations

FL	food literacy
SFLQ	Short Food Literacy Questionnaire
CFA	confirmatory factor analysis
OECD	Organisation for Economic Co-operation and Development
BRIEF	The Brief Health Literacy Screening Tool
FCQ	Food Choice Questionnaire
MBHB model	The Motivators and Barriers of Health Behaviors Model
ICC	Intraclass Correlation Coefficient
CFI	Comparative Fit Index
TLI	Tucker–Lewis Index
RMSEA	Root Mean Square Error of Approximation
SD	standard deviation
